# Global trends in the research on older population dizziness/vertigo: a 20-year bibliometric and visualization analysis

**DOI:** 10.1016/j.bjorl.2024.101441

**Published:** 2024-05-11

**Authors:** Xiang Li, Chao Wei, Xiang Gao, Jie Sun, Jianhong Yang

**Affiliations:** a The First Affiliated Hospital of Ningbo University, Department of Neurology, Ningbo, China; b The First Affiliated Hospital of Ningbo University, Department of Neurosurgery, Ningbo, China

**Keywords:** Dizziness, Vertigo, Older population, Visual analysis, Global trends

## Abstract

**Objective:**

Dizziness or vertigo in older population frequently presents in clinical settings, yet its etiology remains elusive. The objective of this study was to delineate global trends and identify frontiers in research concerning dizziness or vertigo among older population.

**Methods:**

We searched the research literature published from 2003 to 2022 on older population with dizziness or vertigo using two databases from the Web of Science Core Collection. A bibliometric and visualization analysis was conducted. Bibliometric tools facilitated co-authorship, co-citation, and keyword co-occurrence analyses, encompassing countries or regions, institutions, authors, journals, and references.

**Results:**

The analysis included 1322 publications authored by 6524 individuals from 2244 institutions across 67 countries or regions, spanning 92 subject categories. A steady increase in publications was noted from 2003 to 2022. The University of Munich, Harvard University, and the University of California System emerged as leading institutions with the highest publication outputs. The United States, Germany, and China were predominant in publication counts. Eva Grill was identified as the most prolific author. *Otology & Neurotology* and *Geriatrics & Gerontology* emerged as the most prolific journal and subject category, respectively. The most prevalent keywords were “dizziness”, “vertigo”, “falls”, and “geriatric”, with “management”, “gait”, and “association” recognized as the principal research hotspots.

**Conclusion:**

This study provides a systematic analysis of global scientific research on older population dizziness/vertigo, revealing significant advancements in understanding over the past two decades. Management, gait, and association have emerged as the primary research focuses on recent years. These findings offer valuable insights for directing current research efforts to capture prevailing trends and explore new frontiers in this field.

## Highlights

• Dizziness and vertigo are one of the most common complaints in the elderly.
• Management, gait, and association is hot topics in older population dizziness/vertigo.
• The etiology of many older population dizziness/vertigo patients is unknown.
• Unexplained older population dizziness may be associated with cerebral small vessel disease.

## Introduction

Dizziness and vertigo rank among the most prevalent complaints reported by the elderly. Studies indicate that 20%–30% of individuals experience these symptoms.^1^, ^2^, ^3^ The incidence of dizziness increases with age, as evidenced by a cross-sectional Swedish study which found that the prevalence of dizziness in adults over 85 years old rose by approximately 50%.^4^ Typically, benign conditions such as orthostatic hypotension (13%) and peripheral vestibular syndrome (32%) are identified as common causes of these symptoms; however, in about 5% of cases, serious neurological disorders, predominantly cerebrovascular diseases, are diagnosed. Elderly individuals with concurrent dizziness or vertigo are at a heightened risk for falls,^5^, ^6^ representing a significant public health concern. In South Korea, a notable increase in dizziness cases was observed, where the number of affected individuals rose by 4.80% in one year (9,357,233 people, or 7.19% of the population), with healthcare costs surging by 217% to approximately $112,018 million, or about $4 per patient,^7^ placing a substantial strain on the healthcare system.

Dizziness and vertigo disrupt bodily balance, compelling the brain to integrate all available sensory cues from the vestibular, visual, and proprioceptive systems. These inputs are processed by the central nervous system to generate appropriate motor responses. Consequently, lesions in these pathways frequently result in dizziness or vertigo among older population, leading to a multitude of causes including neurological, cardiovascular, visual, vestibular, and psychological factors.^8^ Despite the identification of numerous causes, many instances of these conditions remain unexplained.^9^

This study employs bibliometric methods to investigate older population vertigo and dizziness, aiming to augment current understanding of these conditions. CiteSpace and VOSviewer, extensively used bibliometric tools, effectively visualize the dynamic progression of scientific knowledge, enabling rapid access to pertinent data and providing invaluable guidance.^10^ Beyond simply outlining and predicting the current state, hotspots, and trends within specific research areas through quantitative analysis, bibliometrics also help identify productivity and collaborative patterns among nations, institutions, and authors.^11^ These tools offer critical insights to researchers. Although global studies over the past two decades have clarified mechanisms and treatment strategies for older population dizziness and vertigo, a comprehensive bibliometric analysis of global research trends has yet to be conducted. Therefore, this study hypothesizes that a bibliometric and visual analysis of worldwide research literature from 2003 to 2022 will reveal trends and key areas in older population dizziness and vertigo research, reflecting interdisciplinary research trends and providing a strategic framework and directions for clinicians and researchers to further explore this field.

## Methods

### Data acquisition and search strategy

**Figure 1:**
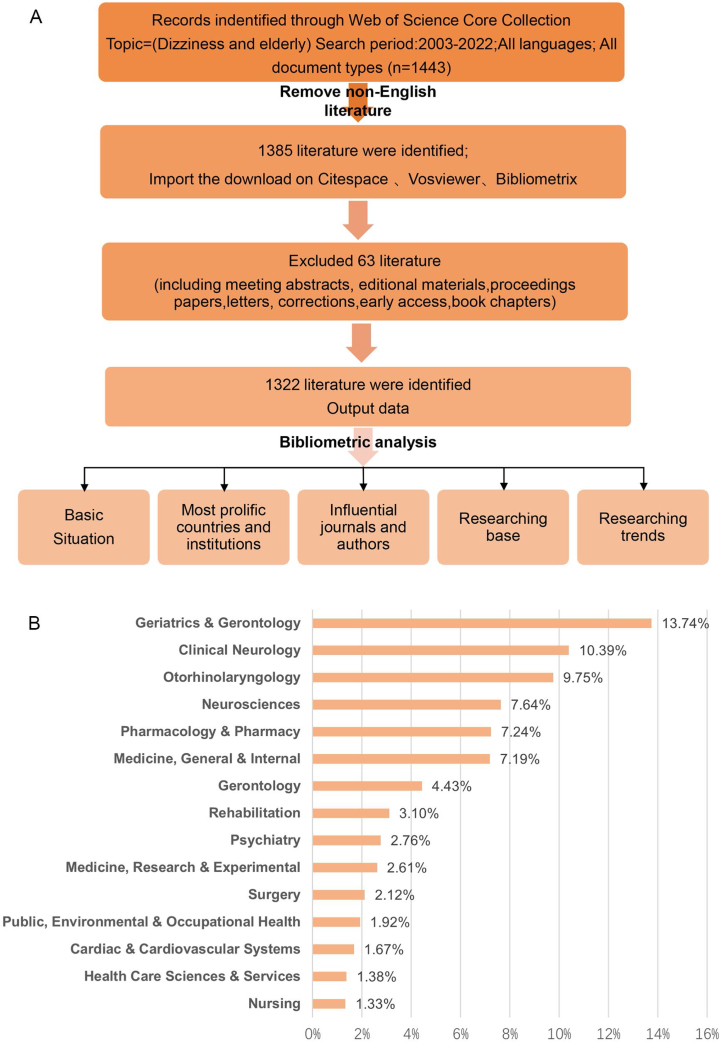
(A) Flow diagram of the publication selection process for a bibliometric analysis. (B) Subject categories of the publications.

This bibliometric analysis was conducted utilizing the Science Citation Index Expanded (SCI-Expanded) and the Social Sciences Citation Index (SSCI) databases from the Web of Science Core Collection. The search criteria were defined as follows: topic = ([Vertigo or Dizziness] and [“elderly patients” or “elderly” or “elderly population” or “aging” or “geriatric”]). The designated study period spanned from January 1, 2003, to December 31, 2022. To ensure the publications were representative of global research trends in older population dizziness/vertigo, only English-language articles and reviews were included. The search yielded records and cited references, which were subsequently downloaded in plain text and tab-delimited formats. The process of selecting publications is illustrated in Fig. 1A. To minimize bias related to routine database updates, all retrievals and downloads were executed on a single day, August 8, 2023. The datasets, consisting of secondary data from the databases devoid of any personal information from individuals, were independently reviewed by two researchers. Given the nature of the data, this study was exempt from requiring informed consent.

### Bibliometric analysis

Publications retrieved from the database were processed using bibliometric analysis software for visualization purposes. The features of these publications, including the countries, institutions, authors, journals, keywords, and references, were systematically catalogued. The impact factors and category quartiles of the journals were extracted from the 2022 Journal Citation Reports. The H-index, a key indicator of scientific impact, was evaluated for various measures related to the study. Microsoft Excel 2021 facilitated the creation of tables and the presentation of publishing trends across global publications.

A total of 1443 publications were retrieved from the Web of Science Core Collection and exported as TXT documents in the “download_xxx” format. A comprehensive data cleaning process was conducted, which involved standardizing English writing styles, merging both full and abbreviated forms of keywords, and reconciling singular and plural forms of keywords. Additionally, synonyms were replaced to eliminate redundancy and maintain the integrity of keyword meanings within the dataset. Microsoft Excel 2021 was employed to log the annual publication volume. The curated literature data underwent visualization analysis using several bibliometric tools. R package (https://bibliometric.com/), VOSviewer 1.6.19 (https://www.vosviewer.com/), CiteSpace 6.2.R3 (https://citespace.podia.com/), and Pajek software (http://vlado.fmf.uni-lj.si/pub/networks/pajek/) were utilized for this purpose. CiteSpace was specifically used to compute the centrality of cooperation networks between institutions and to identify bursts of keywords and citations, which are critical indicators for detecting emerging trends within the field. Meanwhile, VOSviewer facilitated the analysis of cooperation networks among authors, aiding in the identification of key contributors to the field. Additionally, a keyword co-occurrence network was constructed, including only those keywords mentioned more than seven times. VOSviewer assigned these keywords into various clusters based on thematic categories, with different colors representing distinct clusters, thus illustrating the interrelationships among research topics.

CiteSpace, a Java-based visualization tool, was employed to compute centralities and visualize collaborative network graphs for institutions and to map annual citation bursts for keywords and references.^12^ Nodes in these network graphs symbolized the frequency of an item’s occurrence, with nodes of high centrality marked by purple rings, indicating pivotal points within the research domain.^13^, ^14^ The centrality of a node, which increases with the number of links connected to it, was used to gauge the node's importance in the network.

VOSviewer, a contemporary scientometric tool, generated maps from database data and visualized these for analytical review.^15^ In the network visualization diagrams, different clusters were color-coded, with lines between circles indicating collaborative relationships. In temporal visualization charts, varying shades represented different publication years. The network correlation diagrams depicted elements such as countries, institutions, authors, and keywords as circles, with the size of each circle indicating the frequency or quantity of publications or occurrences, and links among circles representing co-authorship, co-occurrence, or co-citation relationships.

## Results

### Analysis of publication output

**Figure 2:**
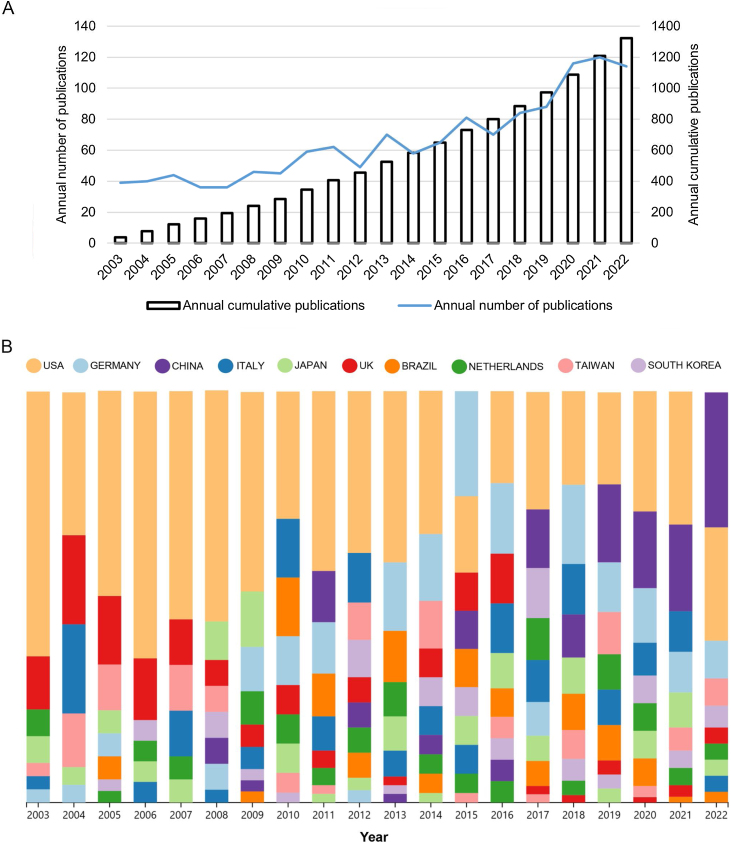
(A) Yearly output of publications from 2003 to 2022. (B) The upward trend in publishing older population dizziness/vertigo related research from 2003 to 2022 among the top 10 countries/regions with the most publications.

The search conducted between 2003 and 2022 retrieved 1322 eligible publications on older population dizziness/vertigo, comprising 1126 original articles and 196 review articles. Each publication was associated with at least one subject category within the Web of Science Core Collection. These publications were distributed across 92 subject categories (illustrated in Fig. 1B). The majority of these publications were in Geriatrics & Gerontology (279 publications), followed by Clinical Neurology (211), Otorhinolaryngology (196), Neurosciences (154), and Pharmacology & Pharmacy (147 articles). A significant upward trend in publication volume is evident, as depicted in Fig. 2A. Notably, a dramatic increase in research activity was observed from 2016 to 2022, during which 559 articles, accounting for more than 50% of the total publications, were produced.

### Distribution by countries and institution

**Figure 3:**
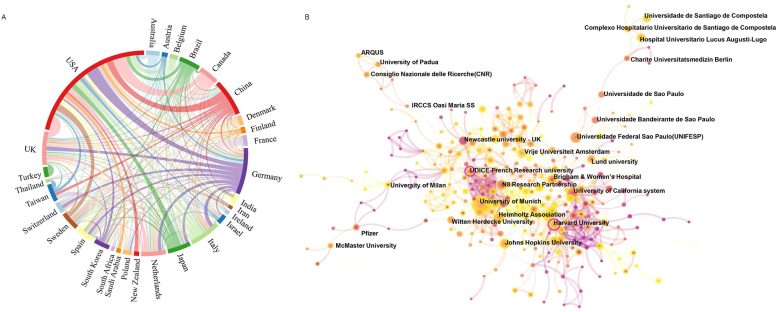
(A) A network map of the cooperative relationship between countries or regions. (B) A visual map of institutions’ contributions to older population dizziness/vertigo research publications.

A total of 1322 papers were published by authors from 67 countries and 2244 institutions. Table 1, Table 2 list the countries and institutions with the highest number of publications, respectively. The United States led in publications with 378 papers, followed by Germany (134), China (106), Italy (90), and the United Kingdom (89) (Table 1). Fig. 2B illustrates an upward trend in publication output among the top 10 countries, indicating changes in the number of papers from these countries in recent years. Fig. 3A displays the cooperative networks among countries; the USA exhibited the highest centrality score (0.47), followed by the UK (0.17), Italy (0.13), Germany (0.12), and China (0.08). An analysis using CiteSpace on the distribution of institutions contributing to the research revealed that the University of Munich was the most productive, publishing 67 papers, followed by Harvard University (29), the University of California System (27), Vrije Universiteit Amsterdam (25), and the N8 Research Partnership (24) (Table 2).

**Table 1:** The top 10 countries with the most publications in the field of older population dizziness/vertigo.

Rank	Country	Count	Centrality
1	USA	378	0.47
2	Germany	134	0.12
3	China	106	0.08
4	Italy	90	0.13
5	UK	89	0.17
6	Netherlands	71	0.01
7	Japan	66	0.01
8	Brazil	61	0.02
9	Canada	57	0.02
10	Spain	53	0.05

**Table 2:** The top 10 institutions with the most publications on older population dizziness/vertigo.

Rank	Country	Country	Count	Centrality
1	University of Munich	Germany	67	0.04
2	Harvard University	USA	29	0.13
3	University of California System	USA	27	0.04
4	Vrije Universiteit Amsterdam	Netherlands	25	0.01
5	N8 Research Partnership	UK	24	0.08
6	Johns Hopkins University	USA	24	0.02
7	Pennsylvania Commonwealth System of Higher Education (PCSHE)	USA	23	0.04
8	US Department of Veterans Affairs	USA	23	0.02
9	Helmholtz Association	Germany	23	0.01
10	University of Pittsburgh	USA	21	0.03

### Distribution by authors

**Figure 4:**
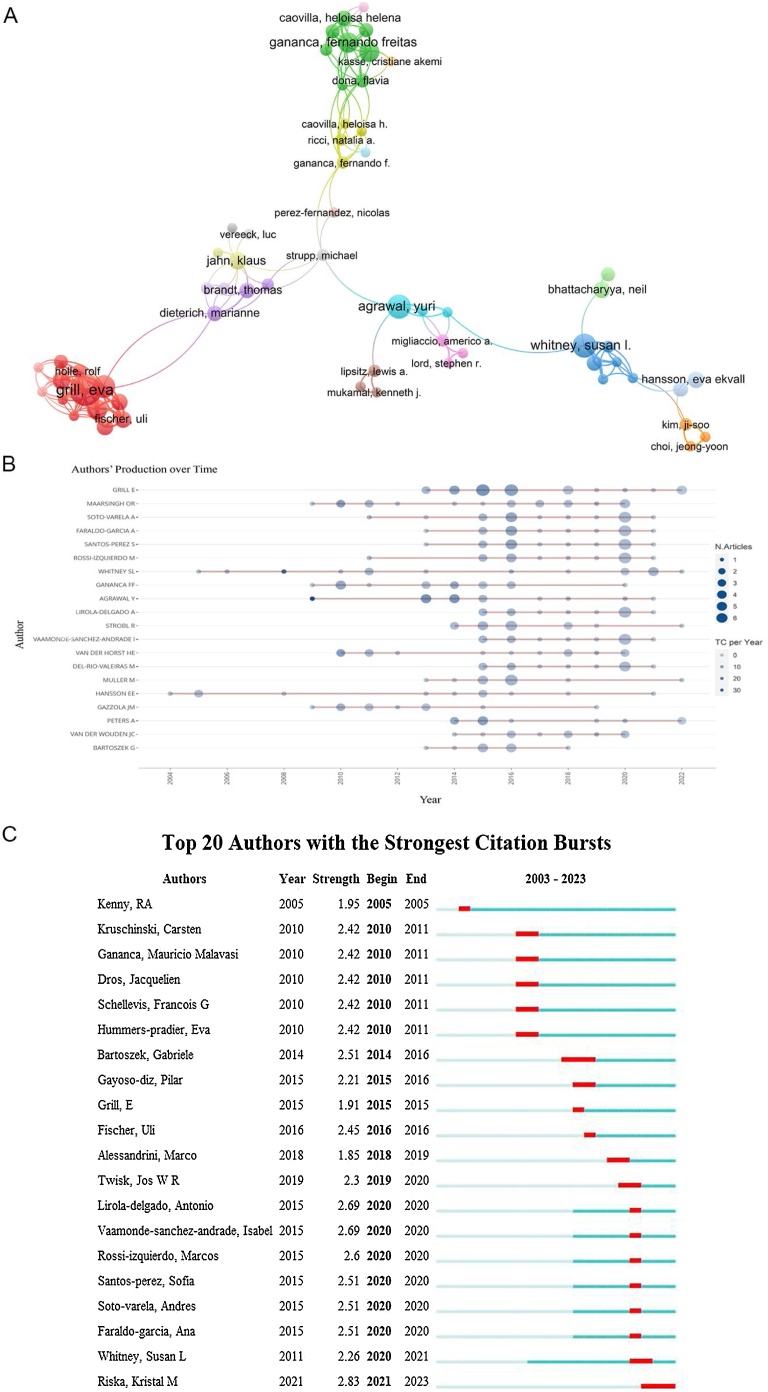
(A) A network visualization map of co-authorship in older population dizziness/vertigo research from 2003 to 2022. (B) The top 20 productive authors in the field over time. (C) The top 20 authors with the strongest citation bursts (2003–2022).

A total of 6524 authors contributed to the field, with the 10 most productive authors listed in Table 3. The collaboration networks among these authors, as shown in Fig. 4A, suggest a fragmented pattern of collaborations, indicating that academic partnerships within this field are relatively limited and infrequent. Fig. 4B displays the top 20 prolific authors at the time of the search. Notably, Eva Grill of Ludwig Maximilians Universität München emerged as the most prolific contributor with 26 publications and 409 citations, followed by Otto R. Maarsingh from the University of Amsterdam with 18 publications and 954 citations, and Andres Soto-Varela from the University of Santiago de Compostela with 17 publications and 231 citations. Eva Grill also achieved the highest H-index of 18 (Table 3). The centrality scores for these authors did not exceed 0.01, indicating a relatively low level of inter-institutional cooperation among academic teams. Fig. 4C presents the top 10 authors with the most significant citation bursts.

**Table 3:** The top 10 authors with the most publications related to older population dizziness/vertigo.

Rank	Author	Count	Country	Centrality	Total number of citations	H-index
1	Grill, Eva	26	Germany	0	409	12
2	Maarsingh, Otto R.	18	Netherlands	0	954	10
3	Soto-Varela, Andres	17	Spain	0	231	8
4	Faraldo-Garcia, Ana	16	Spain	0	175	8
5	Santos-Perez, Sofia	16	Spain	0	175	8
6	Rossi-Izquierdo, Marcos	15	Spain	0	198	8
7	Whitney, Susan L.	15	USA	0	744	8
8	Gananca Ff	14	BRAZIL	0	321	10
9	Van Der Horst, Henriette E.	13	Netherlands	0	328	8
10	Agrawal, Yuri	13	USA	0	954	11

#### Analysis of keywords

**Figure 5:**
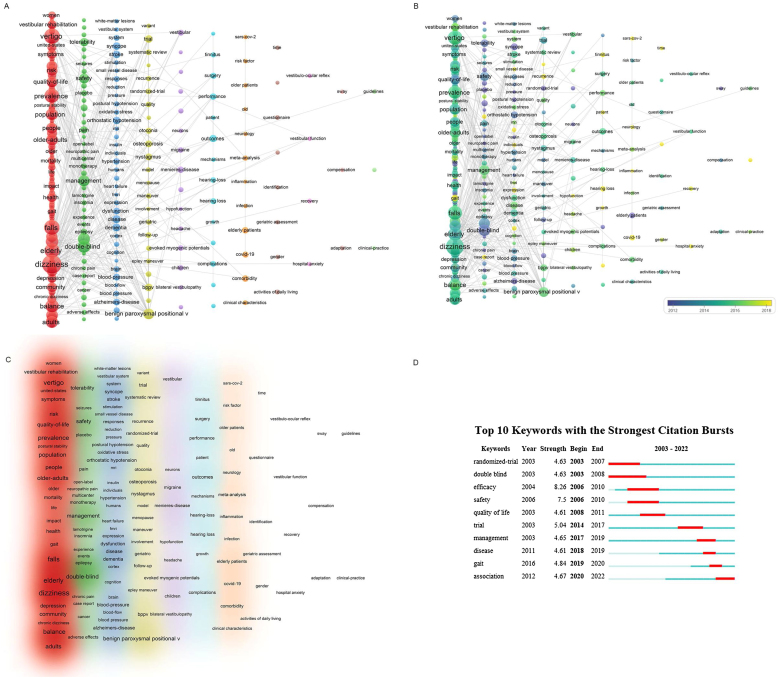
(A) A network diagram of 296 keywords classified into seven clusters. (B) The distribution of keywords is shown in the order of their appearance. (C) The density visualization map of the keywords. (D) The top 10 keywords with the largest citation bursts (2003–2022).

A total of 5511 keywords were retrieved, 296 of which appeared more than seven times (Fig. 5A). The ten most frequent keywords included “dizziness” (318 occurrences), “vertigo” (189), “falls” (174), “elderly” (170), “prevalence” (156), “balance” (129), “double-blind” (117), “elderly-patients” (106), “efficacy” (101), and “adult”. Five thematic clusters were identified: epidemiology of older population dizziness/vertigo, clinical features, etiology, treatment, and clinical research. In the time-overlay network map, keywords were color-coded based on the average year of publication to highlight shifts in focus over time (Fig. 5B). Research prior to 2014 predominantly focused on the epidemiology and clinical characteristics of older population dizziness/vertigo. Recent trends indicate a shift towards exploring concomitant symptoms associated with the condition. The frequency of keyword occurrences determined their density, as depicted in the density graph (Fig. 5C). Keywords exhibiting citation bursts ‒ those frequently cited within specific timeframes ‒ were identified. Fig. 5D lists the top 20 keywords with the most significant citation bursts, sorted by occurrence period. Before 2012, “quality of life” showed the highest burst intensity (4.36), while “trial” exhibited the greatest burst intensity (5) between 2012 and 2019. Post-2019, “ostensible” led in citation bursts, and the keyword “osteoporosis” had the most substantial burst (4.46) after 2019. These data illustrate a dynamic shift in research focus over time, highlighting the evolving nature of scholarly attention within the field.

### Characteristics of the top 10 co-cited publications features of the top ten publications that are co-cited

As detailed in Table 4, the top 10 publications ranked by co-citation numbers collectively received 689 citations, representing 1.67% of the total 41,288 citations recorded. The article “The Development of the Dizziness Handicap Inventory” by Gary P. Jacobson et al., published in 1990 in the *Archives of Otolaryngology-Head & Neck Surgery*, emerged as the most frequently cited work with 105 citations. Among these leading papers, four were published in journals with an impact factor of 10 or higher, namely the *Annals of Internal Medicine*, *Archives of Internal Medicine*, and the *Journal of Neurology, Neurosurgery, and Psychiatry*. Additionally, two were published in journals with impact factors between 5 and 10, specifically *Age and Ageing* and the *Journal of the American Geriatrics Society*. This concise overview highlights the influence and distribution of seminal works within the field as reflected by co-citation metrics, providing insights into the foundational articles driving research on dizziness and vertigo.

**Table 4:** The top 10 co-cited publications related to older population dizziness/vertigo.

Rank	Title	First Author	Year	Journal	Citation
1	The development of the Dizziness Handicap Inventory	Gary P. Jacobson	1990	*Archives of Otolaryngology-Head & Neck Surgery*	105
2	Dizziness among older adults: a possible geriatric syndrome	Mary E. Tinetti	2000	*Annals of Internal Medicine*, 39.2	90
3	Disorders of balance and vestibular function in US adults: data from the National Health and Nutrition Examination Survey, 2001‒2004	Yuri Agrawal	2009	*Archives of Internal Medicine*, 17.33, (Now renamed *JAMA Internal Medicine*)	74
4	“Mini-mental state”. A practical method for grading the cognitive state of patients for the clinician	Marshal F. Folstein	1975	*Journal of Psychiatric Research*, 4.8	73
5	Epidemiology of benign paroxysmal positional vertigo: a population-based study	M von Brevern	2006	*Journal of Neurology, Neurosurgery, and Psychiatry*, 11	69
6	Unrecognized benign paroxysmal positional vertigo in elderly patients	John S. Oghalai	2000	*Otolaryngology-Head and Neck Surgery*, 3.4	64
7	Prevalence of dizziness and vertigo in an urban elderly population	Radi Jönsson	2004	*Journal of Vestibular Research*, 2.3	63
8	The prevalence and characteristics of dizziness in an elderly community	Nicola R. Colledge	1994	*Age and Ageing*, 6.7	55
9	Dizziness: state of the science	Philip D. Sloane	2001	*Annals of Internal Medicine*	49
10	The timed “Up & Go”: a test of basic functional mobility for frail elderly persons	Diane Podsiadlo BScPT	1991	*Journal of the American Geriatrics Society*, 6.3	47

### Analysis of citations

**Figure 6:**
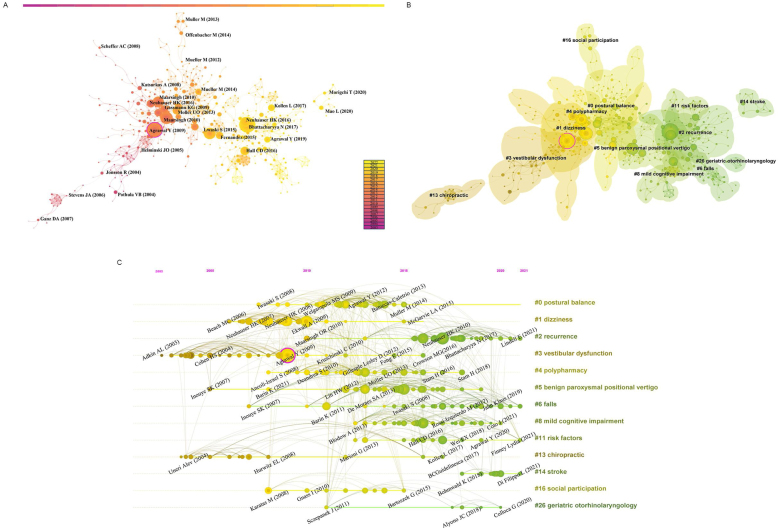
(A) Map of co-cited references of publications related to older population dizziness/vertigo. (B) Cluster analysis of networks with co-cited references. (C) Timeline of co-cited references with clustered labels.

**Figure 7:**
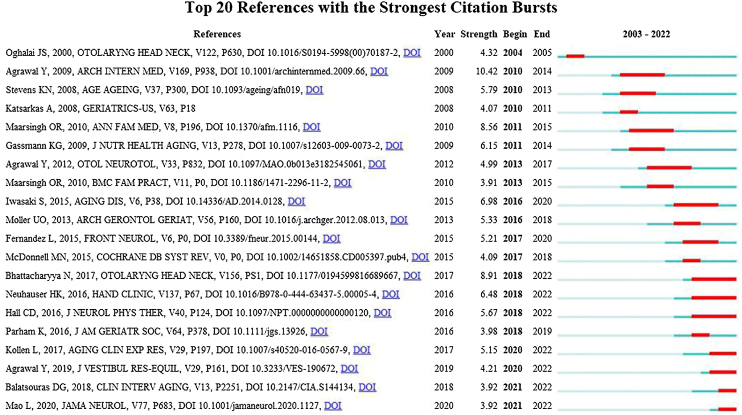
The top 20 references with the largest citation bursts (2003–2022).

A fundamental bibliometric indicator, co-cited references were analyzed from 41,288 references across 1322 publications, resulting in a cluster network diagram featuring 877 nodes and 2093 links (Fig. 6A). The network's thirteen largest clusters are illustrated in Fig. 6B. The largest cluster focused on “postural balance”, followed by “vestibular dysfunction”, “dizziness”, “recurrence”, “benign paroxysmal positional vertigo”, “polypharmacy”, “fall”, “mild cognitive impairment”, “risk factors”, “chiropractic”, “stroke”, “social participation”, and “geriatric otorhinolaryngology”. The roles played by various authors within these research hotspots over time are depicted in Fig. 6C. This timeline visualization (Fig. 6) plots the top 13 clusters, highlighting the scientific applicability of the references within these themes. Fig. 7 showcases the top 20 most cited references, identifying emerging research hotspots in related fields. These references underscore the prominence of older population dizziness/vertigo research within the disciplines of neurology and geriatric medicine.

### Journal source analysis

**Figure 8:**
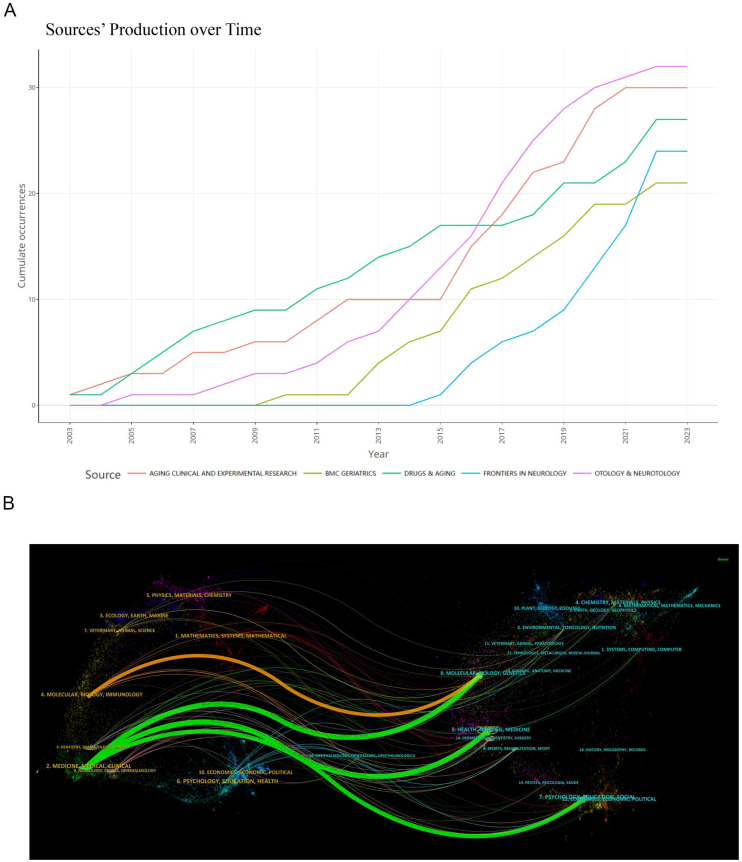
(A) Cumulative publication trends of the top 10 most prolific journals (2003–2022). (B) A dual map overlay of journals related to older population dizziness/vertigo.

A bi-mapped overlay of journal sources and citations is presented in Fig. 8. On the left side of the overlay, journals publishing the studies are depicted, while the right-side displays journals that cite these studies, with routes illustrating the relationships between journal citations. Citation paths are indicated by three green routes and one yellow route. The green routes represent studies published in journals categorized under Medicine, Medical, and Clinical, which were subsequently cited by journals in Health, Nursing, Medicine, Molecular, Biology, Genetics, Psychology, Education, and Social Sciences. The yellow route highlights papers from Molecular, Biology, and Immunology that have been cited by journals in Molecular, Biology, and Genetics. Table 5 lists the ten journals with the highest number of publications, accounting for 16.9% (224 publications) of all studies; 60% of these journals were classified as Q2. It is apparent that research on older population dizziness/vertigo is published across a diverse array of journals, predominantly in Otorhinolaryngology, Neurology, and Geriatrics. However, publications in top-tier journals are less frequent.

**Table 5:** The top-10 most prolific journals for publications related to older population dizziness/vertigo.

Rank	Sources	Count	Country	Journal citation reports (2022)	Impact factors (2022)	Total number of citations	H-Index
1	*Otology & Neurotology*	32	USA	Q3	2.1	698	16
2	*Aging Clinical and Experimental Research*	30	Italy	Q2	4	369	12
3	*Drugs & Aging*	27	New Zealand	Q3	2.8	479	12
4	*Frontiers in Neurology*	24	Switzerland	Q2	3.4	249	8
5	*BMC Geriatrics*	21	England	Q2	4.1	302	9
6	*Clinical Interventions in Aging*	19	New Zealand	Q3	3.6	283	10
7	*Brazilian Journal of Otorhinolaryngology*	18	Brazil	Q2	2.2	269	11
8	*Frontiers in Aging Neuroscience*	18	Switzerland	Q2	4.8	181	8
9	*Journal of the American Geriatrics Society*	18	USA	Q1	6.3	1434	13
10	*Laryngoscope*	17	USA	Q2	2.6	630	14

## Discussion

Dizziness and vertigo in older adults are recognized as escalating public health concerns. Studies indicate that older individuals with these conditions are at an elevated risk of falls, which are the leading cause of hospital admissions and accidental deaths among the elderly. This underscores the necessity of providing a comprehensive overview of the current global research trends in older population dizziness and vertigo. Over the past two decades, the number of publications on older population dizziness and vertigo has consistently increased, highlighting the significant attention that researchers and clinicians are dedicating to this disorder. Advances in laboratory testing techniques suggest that this research area will continue to be a focus in the upcoming decade, likely resulting in a sustained increase in relevant publications.

The United States has emerged as a leader, contributing approximately 29% of global publications in this field. The data reveal that the most prolific institutions are located in developed nations, which significantly advance research in older population dizziness and vertigo. This trend highlights the mature research environments and substantial investments in these countries or regions, reflecting a critical demand for effective treatment and management of older population dizziness and vertigo. Notably, the United States shows the highest level of collaborative exchanges with other nations, securing a top position in centrality scores. China's rapid economic growth has led to a significant increase in publications, making it the country with the most publications on older population dizziness and vertigo by 2022. However, countries like China, the Netherlands, and Canada have low centrality scores, indicating infrequent international collaborations. Encouraging international communication and cooperation, especially among entities utilizing cutting-edge technologies, is recommended for countries with lower centrality scores. The N8 Research Partnership in the UK, Harvard University in the USA, and the University of Munich in Germany are noted for engaging in frequent collaborations, significantly contributing to the development of their respective disciplines. The top three most productive authors ‒ Grill Eva from Germany, Maarsingh Otto R. from the Netherlands, and Soto-Varela Andres from Spain ‒ are based in developed European countries and are affiliated with neurology or geriatrics departments in university hospitals.

Prominent journals that publish articles related to older population dizziness and vertigo include *Otology & Neurotology*, *Aging Clinical and Experimental Research*, *Drugs & Aging*, *Frontiers in Neurology*, and *BMC Geriatrics*. These publications primarily focus on geriatrics, neurology, and otolaryngology, highlighting the significance of older population dizziness within these fields. Although the *Journal of Vestibular Research (JVR)* publishes fewer articles on this topic, it remains a leading journal, known for its contributions to the field, including research papers, review articles, and the development of the International Classification of Vestibular Disorders (ICVD) in collaboration with the Bárány Society. A landmark development in this domain was the 2019 release of the Presbyvestibulopathy (PVP) diagnostic criteria.^16^ These criteria are instrumental in aiding physicians to identify individuals with PVP, thereby facilitating timely interventions such as vestibular rehabilitation. By establishing a standardized approach to diagnosing PVP globally, these guidelines have significantly advanced the study of older population dizziness and vertigo. The Dizziness Handicap Inventory (DHI) scale,^17^ widely cited in the literature from 2003 to 2022, includes 25 sub-items that categorize functional, emotional, and physical impairments associated with dizziness and vertigo. Despite the availability of numerous vestibular assessment tools, the DHI scale remains a fundamental resource for clinicians conducting initial severity assessments and ongoing prognostic evaluations of patients with dizziness and vertigo. Research confirms its strong correlation with vestibular Head Impulse Test (vHIT) gain and the caloric test's CP value.^18^ Visualization of research content distribution reveals clinical medicine and nursing as core disciplines in the study of older population dizziness. Notably, there has been a shift from a single-discipline focus to a more multidisciplinary approach in older population dizziness research, as demonstrated in the visualization chart depicting three primary research pathways. This transition underscores a growing recognition of the complex, multifaceted nature of older population dizziness and the need for an integrated research approach to address it effectively.

Cluster analysis of high-frequency keywords indicates that clinical aspects and etiology of older population dizziness/vertigo continue to be significant topics within the field. Research attention has evolved through distinct phases over the past two decades, marked by themes such as “randomized-trial” (2003–2007), “efficacy” (2006–2010), “quality of life” (2008–2011), “management” (2017–2019), and “association” (2020–2022). This progression reflects a sustained scientific interest in older population dizziness, with initial focuses on impacts on quality of life and management. More recently, there has been a shift towards examining the interconnections of dizziness with other medical conditions. The causes of dizziness among older adults are intricate and multifaceted.^19^ Postural stability is maintained by the integration of somatosensory, visual, and vestibular inputs into the central nervous system, which regulates the musculoskeletal system. Alterations in these contributing factors can lead to dizziness and instability, with potential origins from sensory, visual, vestibular, neural, or muscular sources. Importantly, the functionality of all these components tends to decline with age.^20^ A study involving 9485 elderly Korean patients experiencing dizziness identified diverse diagnoses: 35.8% with peripheral vestibular disease, 19.5% with cerebrovascular disease, 12.5% with psychiatric and functional dizziness disorders, and 5.4% with cardiac disease.^7^ Similarly, research in Germany highlights peripheral vestibular disease as the most prevalent cause of dizziness,^9^ with about 3.7% of patients experiencing unexplained dizziness. Aging is characterized by the simultaneous decline of multiple physiological systems, leading to an expected reduction in vestibular function alongside other sensorimotor, central nervous system, and systemic declines. Studies have shown age-related reductions in vestibular hair cells and neurons, supporting the concept of age-associated vestibular decline. Previous research aligns with these findings, demonstrating declines in semicircular canal function, vestibulocochlear reflex, caloric response, and head thrust dynamic visual acuity.^21^, ^22^, ^23^ Additionally, cervical and ocular Vestibular Evoked Myogenic Potential (VEMP) tests indicate declines in saccular and utricular function with age,^24^, ^25^ explaining why many elderly patients experience chronic dizziness related to age-associated changes in the vestibular system.

Although the etiology of older population dizziness is well-documented, it often remains elusive. Despite comprehensive examinations ‒ including neurologic assessments, vestibular function tests, cardiovascular evaluations, and psychiatric assessments ‒ some cases of dizziness persist without a clear origin, leading to a substantial number of elderly patients experiencing unexplained dizziness or vertigo. Recent studies have revealed a correlation between cerebral Small Vessel white matter Disease (SVD) and unexplained dizziness; cranial MRI scans of patients with unexplained dizziness show severe SVD and associated gait abnormalities.^26^, ^27^ Additionally, central disorders that affect vestibular processing and integration, arising from cortico-subcortical and cortico-cortical interactions, as well as discrepancies between perceived and actual spatial orientations, contribute significantly to 'unexplained' dizziness in the elderly. The subjective perception of balance disturbances is crucial for understanding this clinical syndrome, with patients often reporting, “I have a problem with my balance”, indicating abnormal cortical processing of sensory inputs. Various neural circuits responsible for spatial orientation and motor function are suspected to underlie the manifestation of unexplained dizziness in older adults. An increased burden of SVD might induce microstructural changes in vestibular, balance, or spatial orientation networks, leading to functional remodeling that remains undetected by routine clinical vestibular assessments, which primarily evaluate vestibular-ocular reflex gain. As a result, dizziness is often labeled “unexplained” due to the limitations in detecting these subtle functional changes.^28^ In conclusion, the causes of dizziness among the elderly are complex and multifaceted, as indicated by cluster analysis of co-cited literature. Patients exhibiting dizziness display a spectrum of associated factors such as “falls”, “mild cognitive impairment”, various “risk factors”, and occurrences of “stroke” and other abnormalities. Therefore, it is imperative to adopt a multidisciplinary approach when addressing dizziness in elderly patients. Considering the range of contributing factors from diverse disciplines is pivotal in comprehensively managing senile dizziness.

## Conclusion

Dizziness among the elderly remains a prevalent issue, imposing a significant burden on public health resources due to falls induced by this condition. A systematic analysis of global scientific research trends on older population dizziness has been conducted in this study. Over the past two decades, there has been a substantial advancement in academic understanding of this phenomenon. Recent focal points of research have centered around gait, associations, and case reports pertaining to older population dizziness. These findings delineate the current trajectory and emerging frontiers in this field, guiding ongoing research toward prevailing trends and identifying new areas of focus.

## Funding

This research was supported by Ningbo Top Medical and Health Research Program (nº 2022020304), Traditional Chinese Medicine Science and Technology Project of Zhejiang Province (2021ZA128), Ningbo Public Welfare Research Project (2022S023).

## Conflicts of interest

The authors declare no conflicts of interest.
